# Appendiceal Neuroendocrine Tumor Presenting as Acute Appendicitis: A Case Report

**DOI:** 10.7759/cureus.110881

**Published:** 2026-06-15

**Authors:** Edgar A Flores García, Hector A Lopez Villicaña, Azael Lopez Lopez, José M Hinojosa Rodríguez, Oscar E Coronado Padilla, Abigail Diaz Alba, Héctor X Rodríguez Hernández

**Affiliations:** 1 Surgery, Hospital Nuevo Gómez Palacio, Gomez Palacio, MEX; 2 Surgery, Hospital Nuevo Gomez Palacio, Gómez Palacio, MEX; 3 Surgery, Hospital Nuevo Gómez Palacio, Gómez Palacio, MEX; 4 Surgery, Hospital General de México "Dr. Eduardo Liceaga", Ciudad de México, MEX

**Keywords:** acute abdominal surgery, appendiceal cancer, appendiceal neuroendocrine neoplasm, appendiceal neuroendocrine tumor, atypical appendicitis

## Abstract

Appendiceal neuroendocrine tumors (NETs), historically referred to as carcinoid tumors, are the most common primary neoplasms of the appendix and are frequently diagnosed incidentally following surgery for suspected acute appendicitis. Preoperative diagnosis is uncommon because their clinical presentation often mimics inflammatory appendiceal disease. We report the case of a 30-year-old male who presented with a 48-hour history of right lower quadrant abdominal pain, nausea, vomiting, and fever, consistent with acute appendicitis. Due to clinical suspicion of complicated appendicitis, the patient underwent emergency exploratory laparotomy. Intraoperative exploration revealed a firm heterogeneous mass measuring approximately 6 cm involving the appendix, cecum, and ileocecal valve, prompting en bloc surgical resection with protective ileostomy. Gross examination demonstrated an exophytic appendiceal mass with irregular contours. Histopathological analysis confirmed a well-differentiated appendiceal neuroendocrine tumor with negative surgical margins. Postoperative thoracoabdominopelvic computed tomography showed no evidence of lymph node involvement, local recurrence, or distant metastases. The patient experienced an uneventful postoperative recovery and was referred for oncologic follow-up. This case highlights the importance of considering appendiceal neoplasms in patients presenting with acute appendicitis and underscores the role of intraoperative assessment, histopathological evaluation, and appropriate surgical management in achieving favorable outcomes.

## Introduction

Appendiceal cancer is a rare malignancy, accounting for less than 1% of all gastrointestinal neoplasms, with an estimated incidence that has increased over recent decades [1]. This apparent rise is largely attributed to improved histopathological evaluation and heightened clinical awareness rather than a true increase in disease prevalence [1,2]. Despite this trend, appendiceal tumors remain uncommon and are often underrecognized due to their nonspecific clinical presentation.

The appendix can give rise to a heterogeneous group of neoplasms, including neuroendocrine tumors, mucinous neoplasms, and adenocarcinomas. Among these, neuroendocrine tumors represent the most frequent histological subtype, followed by mucinous appendiceal neoplasms and colonic-type adenocarcinomas [2,3]. Each variant demonstrates distinct biological behavior, prognosis, and therapeutic implications, making accurate histopathological classification essential.

Clinically, appendiceal neuroendocrine tumors most commonly present as acute appendicitis and are frequently diagnosed incidentally following appendectomy performed for presumed benign disease [2]. This presentation is often related to luminal obstruction by the tumor, which may trigger secondary inflammation, bacterial overgrowth, and the same clinical manifestations observed in acute appendicitis. Preoperative diagnosis remains challenging because both clinical findings and imaging features frequently overlap with those of inflammatory appendiceal disease. Consequently, routine histopathological examination of appendectomy specimens remains critical for establishing the diagnosis and guiding further management [3].

Surgical treatment is determined by tumor histology, size, depth of invasion, and the presence of high-risk pathological features. These include tumor size greater than 2 cm, mesoappendiceal invasion, lymphovascular invasion, positive surgical margins, and regional lymph node involvement. While appendectomy alone is often curative for small, well-differentiated neuroendocrine tumors, lesions larger than 2 cm or those exhibiting high-risk features frequently require right hemicolectomy and more extensive oncologic management [3].

Localized well-differentiated appendiceal neuroendocrine tumors are generally associated with an excellent prognosis, with reported five-year survival rates exceeding 95% following complete surgical resection. In contrast, advanced appendiceal malignancies may exhibit more aggressive behavior and require multimodal treatment strategies.

Given the rarity and heterogeneity of appendiceal malignancies, case reports continue to play an important role in improving clinical recognition and reinforcing evidence-based management strategies. The present case highlights the diagnostic challenges and intraoperative decision-making required when a patient with presumed complicated acute appendicitis is found to harbor an unexpected appendiceal neuroendocrine tumor. It also illustrates the management challenges encountered in resource-limited settings where advanced preoperative imaging may not be readily available.

## Case presentation

A 30-year-old male with no significant past medical history presented to the emergency department with a 48-hour history of progressively worsening abdominal pain. The pain initially began in the periumbilical region and subsequently migrated to the right lower quadrant, becoming constant and more intense over time. Associated symptoms included anorexia, nausea, subjective fever, and two episodes of non-bilious vomiting. The clinical picture was highly suggestive of acute appendicitis.

On physical examination, the patient appeared uncomfortable and mildly dehydrated. Vital signs revealed a temperature of 38.2 °C, heart rate of 108 beats/min, blood pressure of 118/72 mmHg, and respiratory rate of 20 breaths/min. Abdominal examination demonstrated localized tenderness in the right iliac fossa with guarding and rebound tenderness. McBurney, Rovsing, and Dunphy signs were positive, consistent with localized peritoneal irritation. Bowel sounds were hypoactive.

Laboratory evaluation demonstrated leukocytosis of 16,400/mm³ with neutrophilic predominance (88%), elevated C-reactive protein of 11.2 mg/dL, and mild hemoconcentration. Renal function and serum electrolytes were within normal limits. Urinalysis showed no evidence of urinary tract infection or hematuria. Given the classic clinical presentation, laboratory findings strongly suggestive of complicated acute appendicitis, and the need for urgent surgical intervention, immediate operative management was prioritized. Furthermore, the patient was treated in a public healthcare institution with limited access to advanced imaging studies in the emergency setting. Consequently, no preoperative computed tomography was obtained, and the patient was taken directly to the operating room.

Given the prolonged duration of symptoms, evidence of systemic inflammatory response, localized peritoneal irritation on physical examination, and a high clinical suspicion of perforated or complicated appendicitis, the patient was taken emergently to the operating room. An exploratory laparotomy through a midline infraumbilical incision was performed to provide adequate exposure and facilitate management of potentially advanced intra-abdominal pathology.

Upon entering the abdominal cavity, moderate inflammatory fluid and multiple dense adhesions involving the cecum, terminal ileum, and adjacent omentum were encountered. Careful dissection of the right lower quadrant revealed a firm heterogeneous mass measuring approximately 6 cm in diameter involving the cecum and ileocecal valve, with marked surrounding inflammatory changes. The appendix could not be clearly identified separately from the lesion.

**Figure 1:**
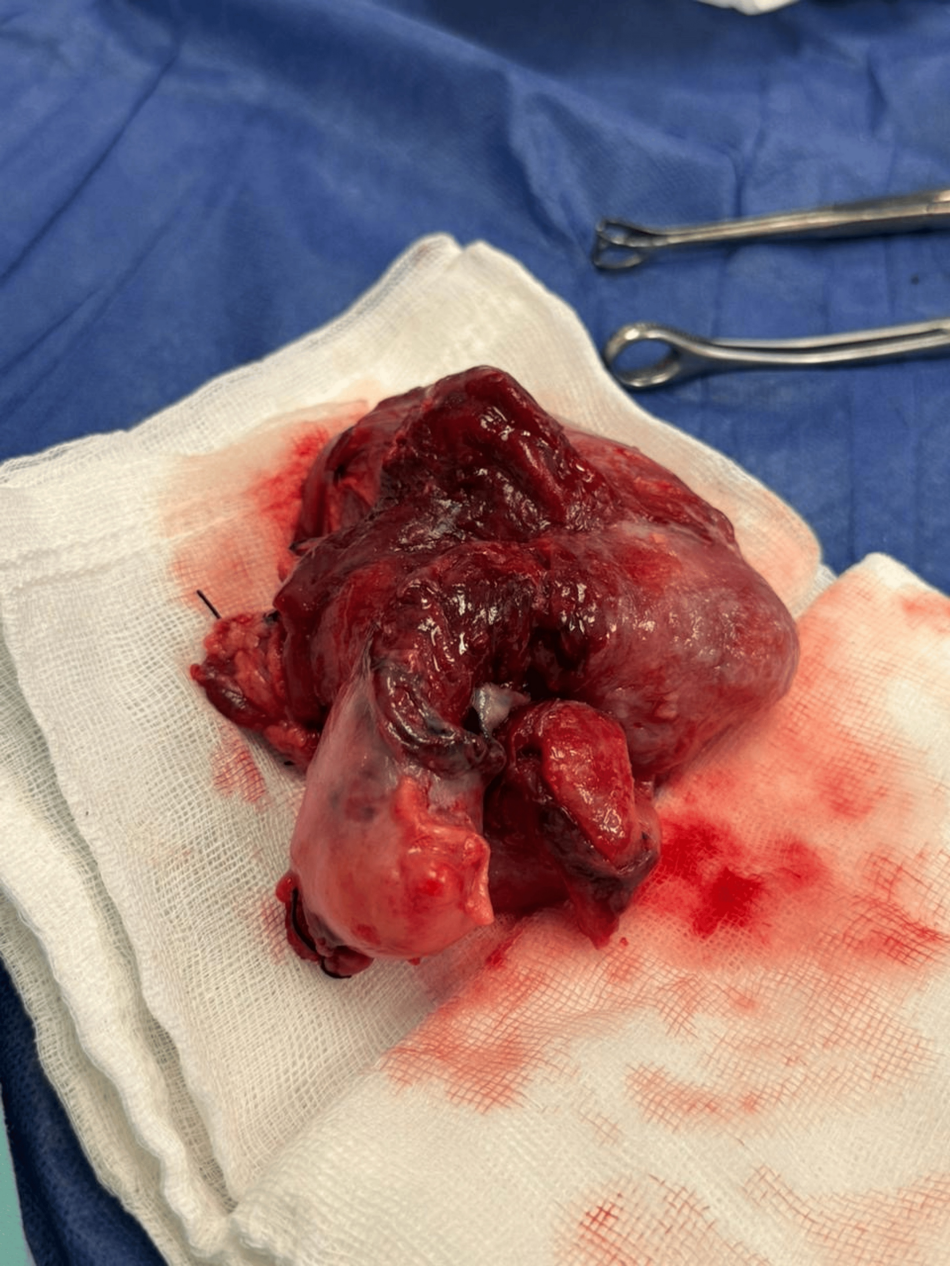
Anterior view of the resected specimen. Gross examination demonstrates an approximately 6-cm exophytic mass arising from the appendiceal region with extension toward the cecum and ileocecal valve. The lesion exhibits irregular borders and a heterogeneous external surface. The specimen was obtained following en bloc resection of the ileocecal segment during emergency surgery.

**Figure 2:**
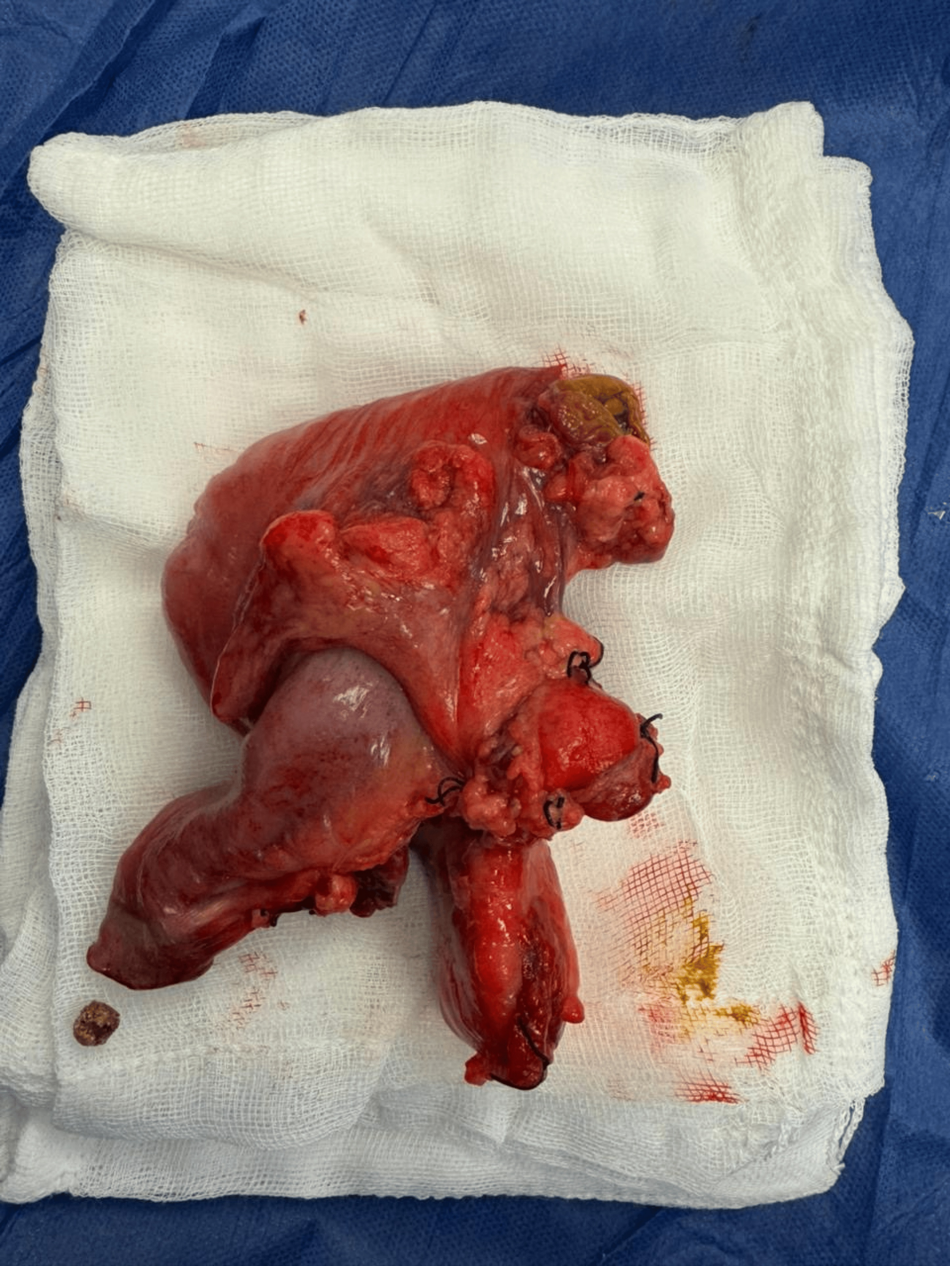
Posterior view of the resected specimen. Posterior aspect of the surgical specimen demonstrating the extent of the appendiceal neuroendocrine tumor and its anatomical relationship to adjacent ileocecal structures. The gross appearance highlights the large size of the lesion and the surrounding inflammatory changes encountered during surgical exploration.

Given the unexpected intraoperative findings and concern for a complicated neoplastic process, en bloc surgical resection was undertaken (Figures 1, 2). The procedure consisted of a right hemicolectomy including the cecum, appendix, ileocecal valve, and approximately 15 cm of terminal ileum proximal to the ileocecal junction. A protective ileostomy was created at the discretion of the operating team due to the extent of resection and local inflammatory changes. The abdominal cavity was thoroughly irrigated, hemostasis was achieved, and the surgical specimen was submitted for histopathological examination.

Histopathological examination revealed a well-differentiated Grade 2 appendiceal neuroendocrine tumor. The lesion demonstrated a Ki-67 proliferation index of 14% and a mitotic count of 12 mitoses per 10 high-power fields (HPF). Immunohistochemical staining was positive for synaptophysin and chromogranin A (++), confirming neuroendocrine differentiation. Surgical margins were free of tumor, indicating complete resection. Detailed information regarding the depth of invasion, mesoappendiceal involvement, and TNM staging was not available in the original pathology report. The postoperative course was uneventful.

As part of postoperative oncologic staging, a contrast-enhanced thoracoabdominopelvic computed tomography scan was obtained to evaluate for metastatic disease. Imaging demonstrated no evidence of local recurrence, lymph node involvement, or distant metastases. The patient was subsequently referred for continued oncologic follow-up.

## Discussion

Appendiceal neuroendocrine tumors (NETs), historically referred to as carcinoid tumors, represent the most common primary malignancy of the appendix and are frequently diagnosed incidentally after appendectomy performed for suspected acute appendicitis [4]. Their clinical presentation is typically nonspecific, and preoperative diagnosis remains uncommon, particularly in young patients with classic signs of appendicitis, as illustrated in the present case.

Tumor size, depth of invasion, and involvement of adjacent structures are key prognostic factors in appendiceal NETs and play a central role in guiding surgical management [5,6]. Lesions smaller than 1 cm confined to the appendix are generally treated with appendectomy alone, while tumors larger than 2 cm or those involving the base of the appendix, mesoappendix, or regional lymph nodes are associated with a higher risk of metastasis and often warrant more extensive surgical resection [6]. In this case, the intraoperative finding of a 6 cm mass involving the ileocecal valve justified an oncologic resection rather than simple appendectomy.

Right hemicolectomy is commonly recommended for large appendiceal NETs or when high-risk features are present, as it allows for adequate lymphadenectomy and margin control [6,7]. The extent of surgical resection in our patient was dictated by the unexpected intraoperative discovery of a sizable tumor with cecal involvement, leading to en bloc resection and protective diversion. Complete surgical excision with negative margins remains the most important determinant of a favorable outcome in localized disease [7].

Postoperative staging with cross-sectional imaging is essential to exclude metastatic disease, particularly to the liver and regional lymph nodes, which are the most common sites of spread in advanced appendiceal NETs [8]. In the present case, thoracoabdominopelvic computed tomography revealed no evidence of metastatic disease, supporting the diagnosis of localized disease and reinforcing the excellent prognosis associated with complete resection.

Although appendiceal NETs are generally indolent, long-term follow-up is recommended, especially in patients with large tumors or atypical features, due to the potential for delayed recurrence [9]. Multidisciplinary evaluation is crucial to ensure appropriate surveillance and to determine the need for additional oncologic therapy, which is typically reserved for metastatic or unresectable disease [10].

This case highlights the diagnostic challenges associated with appendiceal malignancies and underscores the importance of careful intraoperative assessment and routine histopathological examination of appendectomy specimens. Early recognition and individualized surgical management are essential to optimize outcomes in patients with this rare entity.

Although contrast-enhanced computed tomography is currently considered the preferred imaging modality for evaluating suspected complicated appendicitis and may facilitate identification of underlying appendiceal neoplasms, access to advanced imaging remains limited in some healthcare settings. In the present case, the diagnosis was established intraoperatively because the clinical presentation was indistinguishable from complicated acute appendicitis, and preoperative CT imaging was not available.

In the present case, the tumor measured approximately 6 cm and involved the ileocecal region. This size substantially exceeds the 2-cm threshold commonly used to identify patients at increased risk of nodal metastasis and to guide consideration of right hemicolectomy. Consequently, the intraoperative decision to perform an oncologic resection was consistent with current recommendations for large appendiceal neuroendocrine tumors.

Although the diagnosis of a Grade 2 neuroendocrine tumor was established, detailed pathological staging information, including depth of invasion and AJCC/TNM classification, was not available in the original pathology report. This represents a limitation of the present case report.

## Conclusions

This case highlights the diagnostic challenge posed by appendiceal neuroendocrine tumors presenting as acute appendicitis, particularly in young patients without clinical features suggestive of malignancy. Despite a classic presentation of complicated appendicitis, intraoperative exploration revealed a large 6-cm appendiceal mass involving the ileocecal region, requiring right hemicolectomy and protective ileostomy. Histopathological examination confirmed a Grade 2 well-differentiated neuroendocrine tumor with negative surgical margins, and postoperative imaging demonstrated no evidence of metastatic disease. This report underscores the importance of maintaining awareness of underlying appendiceal neoplasms in patients with presumed appendicitis and highlights the role of intraoperative judgment in guiding appropriate oncologic resection when unexpected findings are encountered.

## References

[1] Marmor S, Portschy PR, Tuttle TM, Virnig BA (2015). The rise in appendiceal cancer incidence: 2000-2009. J Gastrointest Surg.

[2] Connor SJ, Hanna GB, Frizelle FA (1998). Appendiceal tumors: retrospective clinicopathologic analysis of appendiceal tumors from 7,970 appendectomies. Dis Colon Rectum.

[3] Kelly KJ (2015). Management of appendix cancer. Clin Colon Rectal Surg.

[4] McCusker ME, Coté TR, Clegg LX, Sobin LH (2002). Primary malignant neoplasms of the appendix: a population-based study from the surveillance, epidemiology and end-results program, 1973-1998. Cancer.

[5] Carr NJ, Cecil TD, Mohamed F (2016). A consensus for classification and pathologic reporting of pseudomyxoma peritonei and associated appendiceal neoplasia: the results of the Peritoneal Surface Oncology Group International (PSOGI) Modified Delphi Process. Am J Surg Pathol.

[6] Pape UF, Niederle B, Costa F (2016). ENETS consensus guidelines for neuroendocrine neoplasms of the appendix (excluding Goblet cell carcinomas). Neuroendocrinology.

[7] Shaib WL, Assi R, Shamseddine A (2017). Appendiceal mucinous neoplasms: diagnosis and management. Oncologist.

[8] Turaga KK, Pappas SG, Gamblin T (2012). Importance of histologic subtype in the staging of appendiceal tumors. Ann Surg Oncol.

[9] Smeenk RM, van Velthuysen ML, Verwaal VJ, Zoetmulder FA (2008). Appendiceal neoplasms and pseudomyxoma peritonei: a population based study. Eur J Surg Oncol.

[10] Overman MJ, Fournier K, Hu CY (2013). Improving the AJCC/TNM staging for adenocarcinomas of the appendix: the prognostic impact of histological grade. Ann Surg.

